# Heterozygous *CYP27B1* c.262delG pathogenic variant and its impact on vitamin D metabolites and phosphocalcic profile in humans

**DOI:** 10.3389/fphys.2025.1716877

**Published:** 2026-01-07

**Authors:** Lysanne Girard, Carol-Ann Fortin, Rosalie Plourde, Véronique Desgagné, Renée Guérin, Caroline Albert, Mathieu Desmeules, Patrice Perron, Luigi Bouchard

**Affiliations:** 1 Department of Biochemistry and Functional Genomics, Faculty of Medicine and Health Sciences, Université de Sherbrooke, Sherbrooke, QC, Canada; 2 Centre de recherche et d’innovation, Centre intégré universitaire de santé et de services sociaux (CIUSSS) du Saguenay-Lac-Saint-Jean, Saguenay, QC, Canada; 3 Department of Biochemistry, Microbiology and Bioinformatics, Faculty of Science and Engineering, Laval University, Québec City, QC, Canada; 4 Clinical Department of Laboratory Medicine, Centre intégré universitaire de santé et de services sociaux (CIUSSS) du Saguenay-Lac-Saint-Jean - Hôpital de Chicoutimi, Saguenay, QC, Canada; 5 Clinical Biochemistry Service, CHUM (Centre hospitalier de l’Université de Montréal), Montreal, QC, Canada; 6 Division of Medical Genetics, Department of Pediatry, Faculty of Medicine and Health Sciences, University of Sherbrooke, Sherbrooke, QC, Canada; 7 Department of Pediatry, CIUSSS du Saguenay-Lac-Saint-Jean, Saguenay, QC, Canada; 8 Department of Medicine, Faculty of Medicine and Health Sciences, Université de Sherbrooke, Sherbrooke, QC, Canada; 9 Centre de Recherche du Centre Hospitalier Universitaire de Sherbrooke, Sherbrooke, QC, Canada

**Keywords:** bone remodeling, calcitriol, *CYP27B1* gene, genetic variant, heterozygocity, parathormone (PTH), phosphocalcic balance, vitamin D-dependent rickets type 1A

## Abstract

**Background/aims:**

Vitamin D-dependent rickets type 1A is a rare autosomal recessive disorder caused by pathogenic variants in the *CYP27B1* gene. *CYP27B1* encodes for the 1α-hydroxylase enzyme catalyzing the conversion of 25-hydroxyvitamin D (25(OH)D) to calcitriol, the last step of vitamin D activation. In the Saguenay–Lac-Saint-Jean (SLSJ) region (Quebec, Canada), the *CYP27B1* c.262delG variant has a carrier rate of 1 in 27 due to a founder effect. This study aimed to characterize the impact of heterozygosity for the *CYP27B1* c.262delG variant on vitamin D metabolites and the phosphocalcic profile.

**Methods:**

Participants from SLSJ were recruited by telephone (n = 36). During an in-person visit, buccal swabs, blood samples, and health and lifestyle information were collected. The *CYP27B1* c.262delG variant was genotyped using TaqMan assays on DNA from buccal swabs, and participants were grouped as carriers (heterozygous for the variant) and non-carriers. Student’s t-test was applied to compare vitamin D metabolites (25(OH)D and calcitriol), parathormone, alkaline phosphatase (ALP), bone alkaline phosphatase (BAP), ionized calcium, and inorganic phosphorus blood levels between carriers and non-carriers.

**Results:**

Carriers showed significantly higher levels of parathormone, ALP, and BAP compared to non-carriers (p < 0.05). Additionally, 25(OH)D levels were higher in carriers, although the difference did not reach nominal statistical significance (p = 0.056). Calcitriol, ionized calcium, and inorganic phosphorus levels were similar between groups.

**Conclusion:**

Heterozygosity for *CYP27B1* c.262delG leads to changes on the vitamin D metabolites and the phosphocalcic profile. How these changes impact the risk of other vitamin D deficiency-associated conditions remain unknown.

## Introduction

Vitamin D-dependent rickets type 1A (VDDR1A) is a rare autosomal recessive disorder caused by biallelic pathogenic variants in the cytochrome P450 family 27 subfamily B member 1 gene (*CYP27B1*). In the Saguenay–Lac-Saint-Jean (SLSJ), a region of Quebec, Canada, VDDR1A is highly prevalent due to a founder effect (Fortin et al., 2025). To date, all molecularly confirmed cases of VDDR1A in the SLSJ region have been attributed to a single pathogenic variant, NM_000785.4(CYP27B1):c.262delG (p.Val88Trpfs*71) (VCV000001664.9-ClinVar-NCBI, 2025), with a carrier frequency we have estimated to 1 in 27 (Fortin et al., 2025). This results in one affected child born annually (prevalence of 1/2916) in this region (Fortin et al., 2022). This variant was also recently (1/25) found to be frequent in the Beauce region of Quebec, Canada (Gagnon et al., 2025). The *CYP27B1* gene encodes for 1-α-hydroxylase, a renal enzyme that catalyzes the last step of vitamin D activation (Marcelli, 2012). The c.262delG (p.Val88Trpfs*71) pathogenic variant causes a frameshift from codon 88 followed by a premature stop at codon 159, resulting in a significantly truncated protein and an almost complete loss of enzymatic activity (Yoshida et al., 1998). This variant thus prevents the conversion of 25-hydroxyvitamin D (25(OH)D) into 1,25-dihydroxyvitamin D (calcitriol) and is associated with a drop of blood calcitriol levels in homozygous individuals. Calcitriol regulates calcium and phosphate homeostasis by enhancing intestinal absorption and promoting bone remodeling (Afzal et al., 2021; Rodriguez et al., 2014). Its synthesis in the kidney is regulated by phosphocalciotropic hormones, such as parathormone (PTH), which stimulates 1-α-hydroxylase activity (Fraser and Kodicek, 1970; Brunette et al., 1978), and fibroblast growth factor 23 (FGF-23), which inhibits 1-α-hydroxylase activity and promotes calcitriol degradation via *CYP24A1* (Blau and Collins, 2015). Calcitriol also downregulates its own synthesis through feedback inhibition, as well as by extracellular calcium and phosphate. Low calcium and phosphate concentrations stimulate 1-α-hydroxylase activity (Massart et al., 2012; Souberbielle, 2012).

Individuals with VDDR1A present a dysregulated phosphocalcic profile within the first week after birth (Fortin et al., 2022; Acar et al., 2018). However, the impacts of carrying only one pathogenic allele of the *CYP27B1* c.262delG variant is currently unknown but could be important for prevention of osteoporosis and other vitamin D deficiency associated conditions in regions with high prevalence of these disorders such as the SLSJ. Accordingly, this study aims to assess the impacts of heterozygosity for a nonsense and loss of function-associated variant (c.262delG) in the *CYP27B1* gene on the vitamin D metabolites and phosphocalcic profiles in human.

## Methods

### Selection of the study participants

Participants were recruited in the SLSJ region (Quebec, Canada) and attended in an in-person visit to the *Centre d’études cliniques du CIUSSS-SLSJ* between July 6th and 15 August 2023. A flowchart detailing the selection of study participants is presented in Supplementary Figure S1. Initial contact was carried out by telephone by a trained research staff and included only persons of legal age and capacity. We first selected parents who agreed to be recontacted as part of a prior research project we have conducted (Fortin et al., 2022) and having a child heterozygous or homozygous for the c.262delG variant of the *CYP27B1* gene (n = 95 families). From these, 29 families were excluded: one parent is affected with VDDR1A and confirmed to be homozygous for the pathogenic variant in the gene *CYP27B1* and 28 families were considered living far from the study site (more than 80 km/90 min drive). Sixteen families could not be reached (wrong number or number out of service (n = 6) or no answer (n = 10)). From the 50 families successfully contacted, only 11 accepted to participate; 6 with parental dyad (n = 12) and 5 with one parent only (n = 5). To increase the sample size, we contacted parents of a child diagnosed with VDDR1A followed at the Metabolic Disease Clinic of the *Centre Intégré Universitaire de Santé et de Services Sociaux du SLSJ* (CIUSSS-SLSJ; n = 10 parents, 7 of whom agreed to participate in the study), and recruited members of research staff and their relatives (n = 12). A total of 36 participants were included in the study. An information and consent form (ICF) was presented to all participants by a trained research staff to obtain their informed consent to participate in the study. This study was approved by the CIUSSS-SLSJ Ethic Research Board.

### Anthropometric measures

Weight, height and waist circumference were measured using standardized procedures during an in person visit to the *Centre d’études cliniques du CIUSSS-SLSJ*. Body mass index (BMI) was then calculated using the formula BMI = weight (kg)/height (m)^2^. We also measured body composition (fat mass, fat free mass and bone mass) by bioelectrical impedance (Tanita, model SC-331S).

### Health and lifestyle questionnaire

Assisted by a research staff, each participant completed a health and lifestyle questionnaire collecting general information on the participant (age, sex, ethnicity, ancestry), medical history (known diagnosis), medication (all regularly prescribed drugs, including dosage, and frequency and vitamin D supplementation), lifestyle habits (alcohol, tobacco and cannabis use as well as sun exposure) and physical activities (type and intensity of physical activity (light, moderate, or vigorous) practiced for at least 30 consecutive minutes over the past 7 days). All answers were self-reported by the participants and provided information on variables potentially influencing the vitamin D metabolism and phosphocalcic profile.

### Buccal cells collection and genetic analysis

The carrier status for the c.262delG variant of the *CYP27B1* gene for all participants was assessed by genotyping as detailed previously in Fortin et al. (2022). Briefly, the buccal cell samples were collected using a self-collection kit during the in person visit to the *Centre d’études cliniques du CIUSSS-SLSJ*. For each participant, two samples were collected, one from the inside of each cheek, using cotton swabs (Puritan, cat # 25-806 1 WC EC). DNA from buccal cells was then extracted using the *DNA Extract All Reagents Kit* (ThermoFisher Scientific, cat # 4403319), following the manufacturer procedure. Genotyping of the c.262delG variant of the *CYP27B1* gene is based on nucleic acid amplification by PCR and TaqMan chemistry, using two sets of specific TaqMan primers and probes previously designed and clinically validated by our team. The genotyping analyses were performed on the 7500 Fast Real-Time PCR thermal cycler from Applied Biosystems. Individuals heterozygous for the *CYP27B1* c.262delG variant were grouped as carriers, whereas individuals for whom the variation was not detected were grouped as non-carriers.

### Blood sampling

Fasting blood samples (8 h) were collected for each participant by trained research nurses of the *Centre d’études cliniques du CIUSSS-SLSJ.* Two vacutainer tubes containing EDTA (Fisher Scientific; cat # 02-683-99C) and three vacutainer serum separation tubes (Fisher Scientific; cat # 02-683-97) were collected for each participant. The serum separation tubes were incubated for 30 min at room temperature, then centrifuged for 10 min at 2,000 g to isolate serum. Two vacutainer tubes containing EDTA were used immediately after sampling (fresh) for the complete blood count and glycated hemoglobin measurement, as well as one vacutainer serum separation tube for ionized calcium measurement. An aliquot of serum was conserved in a cryovial at −80 °C before being shipped to the *Centre hospitalier de l’Université de Montréal* (CHUM) on dry ice for the calcitriol and BAP measurements. Remaining serum aliquots were kept at −20 °C until analysis (PTH, ALP, inorganic phosphorus, lipid profile, glucose and 25(OH)D, FGF-23).

### Biochemical measurement of vitamin D, its metabolites, and assessment of phosphocalcic profile

Blood sample analyses included a complete blood count (Beckman Coulter DXH900), a lipid profile (High-Density Lipoprotein Cholesterol (HDL-C; Beckman Coulter AU5800; cat # OSR6295), Low-Density Lipoprotein Cholesterol (LDL-C; Beckman Coulter AU5800; cat # OSR6196), Total-Cholesterol (Total-C; Beckman Coulter AU5800; cat # OSR6216) and Triglycerides (TG; Beckman Coulter AU5800; cat # OSR6118)), glycated hemoglobin (Sebia Capillarys 3TERA; cat # CAPI3 HbA1c: 2515), phosphocalcic profile (PTH (Beckman Coulter DXI800; cat # A16972), ionized calcium (Radiometer ABL800; cat #variable), ALP (Beckman Coulter AU5800; cat # OSR6004) and inorganic phosphorus (Beckman Coulter AU5800; cat # 6122 OSR)), 25(OH)D (Beckman Coulter DXI800; cat # A98856) and glucose (Beckman Coulter AU5800; cat # OSR6221) levels and were performed at the certified Clinical Biochemistry Laboratory, Chicoutimi Hospital, under the supervision of a certified clinical biochemist. The calcitriol (Diasorin Liaison XL; cat # 310980) and bone alkaline phosphatase (BAP) (Beckman Coulter Dxi 800; Ostase; cat # 37300) measurements were performed at the certified CHUM’s laboratory, also under the supervision of a certified clinical biochemist. FGF-23 measurement was performed in the research laboratory by trained staff using the Human FGF-23 ELISA Kit (Thermofisher Scientific; #cat EH189RB) and standard manufacturer’s protocol and results were imaged using the absorbance mode (450 nm) on the Agilent BioTek Synergy LX multi-mode plate reader.

### Statistical analysis

Normality was first assessed for each variable using the Shapiro-Wilk test. All variables, except ALP, BAP and 25(OH)D, followed a normal distribution. To normalize the distribution, the ALP, BAP and 25(OH)D concentrations were transformed in natural log (ln). The variance homoscedasticity between the carrier group and the non-carrier group for each variable was confirmed using the Bartlett test. We then performed t-tests to compare the means between the *CYP27B1* c.262delG carrier and non-carrier groups. We further applied a Benjamini–Hochberg adjustment on the p-values (p_adj_) to account for multiple-testing. For categorical variables, we used Pearson’s chi-squared (Chi^2^) test when the expected cell counts were higher than 5, and Fisher’s exact test when sample sizes were small or expected counts were lower than 5 to assess differences between the groups. To control for potential cofounding factors, we adjusted the statistical models for (1) age, (2) sex, (3) BMI, (4) age, sex and BMI combined and (5) self-reported vitamin D consumption and sun exposure/protection data (vitamin D supplementation, portions of fish per week, use of sunscreen, wearing long clothing for sun protection, restriction of outdoor activities, avoidance of sun exposure between 10 a.m. and 2 p.m., skin pigmentation, vacation in a sunny location during the last 4 months, and use of tanning salon) in an analysis of covariance (ANCOVA). We also performed sex-stratified analyses to assessed if the biological differences between females and males influence biomarker levels and impact the differences we observed between the non-carriers and the carriers of the c.262delG pathogenic variant. To evaluate whether our study was sufficiently powered, we computed the effect size (Hedge’s g, that accounts and corrects for small sample size) for each of the tested variables. Statistical analyses were performed using R vesion 4.5.1 (2025-06-13) in RStudio version 2025.05.1-513.

## Results

### Participant’s characteristics

Characteristics of the 36 men and women recruited in our study are shown in Table 1 by *CYP27B1* c.262delG variant carrier status: 19 participants (52.8%) were non-carriers, and 17 participants (47.2%) were carriers. No statistical difference was found for all characteristics between the groups (p > 0.05). Briefly, members of the carrier group (36 ± 5 years; mean standard deviation (SD)) were on average slightly older than those of the non-carrier group (32 ± 8 years). In the carrier group, 53% were male, compared to 32% in the non-carrier group. Self-reported ethnicity and SLSJ ancestry showed that 88% of the carrier group were of European descent, and 94% had at least one grandparent from the SLSJ region. For the non-carrier group, 100% reported to be of European descent, with 79% of SLSJ ancestry. On average, the carrier group had a slightly higher BMI and body fat (%) level (carriers: mean BMI of 30.95 ± 5.67 kg/m^2^ and body fat percentage of 34.22% ± 10.12%; non-carriers: mean BMI of 27.27 ± 6.08 kg/m^2^ and mean body fat percentage of 30.53% ± 9.54%) and bone mass (carriers: 2.98 ± 0.62 kg; non-carriers: 2.67 ± 0.54 kg) as compared to non-carriers. None of these differences reached statistical significance (p > 0.05). Other information reported by the participant in the questionnaire that could potentially affect vitamin D metabolism and phosphocalcic profile, including medical history, medication and lifestyle habits, are shown in Supplementary Table S1. Briefly, the data reported by participants indicates that there are no difference between the two groups regarding their consumption habits (vitamin D supplementation, alcohol, tobacco, cannabis, portions of fish per week and vitamin D supplementation), their sun exposure and protection habits (sunscreen, avoiding sun between 10 a.m. and 2 p.m., hat/long clothing, restriction of outdoor activities, vacation in a sunny location in the last 4 months, tanning salon), their skin tone, and their physical activity levels (at least 30 consecutive minutes over the past 7 days) (p > 0.05).

**TABLE 1:** Participant’s characteristics.

Characteristics	Non carriers (n = 19)		Carriers (n = 17)		Student’s t-test/Chi^2^/Fisher p-value
	Mean ± standard deviation	Range	Mean ± standard deviation	Range	
	Number (%)		Number (%)		
Age (years)	32 ± 8	23–49	36 ± 5	26–49	p = 0.09^†^
Sex					
Male	6 (32%)		9 (53%)		p = 0.3
Female	13 (68%)		8 (47%)		
Ethnicity					
European descent	19 (100%)		15 (88%)		p = 0.2
Other	0 (0%)		2 (12%)		
SLSJ ancestry					
Yes	15 (79%)		16 (94%)		p = 0.3
No	4 (21%)		1 (6%)		
Body Mass index (kg/m^2^)	27.27 ± 6.08	16.98–43.11	30.95 ± 5.67	22.52–44.45	p = 0.07†
Body fat (%)	30.53 ± 9.54	18.00–51.80	34.22 ± 10.12	18.00–48.10	p = 0.3
Bone Mass (kg)	2.67 ± 0.54	2.00–3.70	2.98 ± 0.62	2.10–4.30	p = 0.1

Abbreviations: SLSJ, Saguenay-Lac-Saint-Jean.

^†^p < 0.1.

### Comparison of vitamin D and phosphocalcic profile between carriers and non-carriers

**FIGURE 1:**
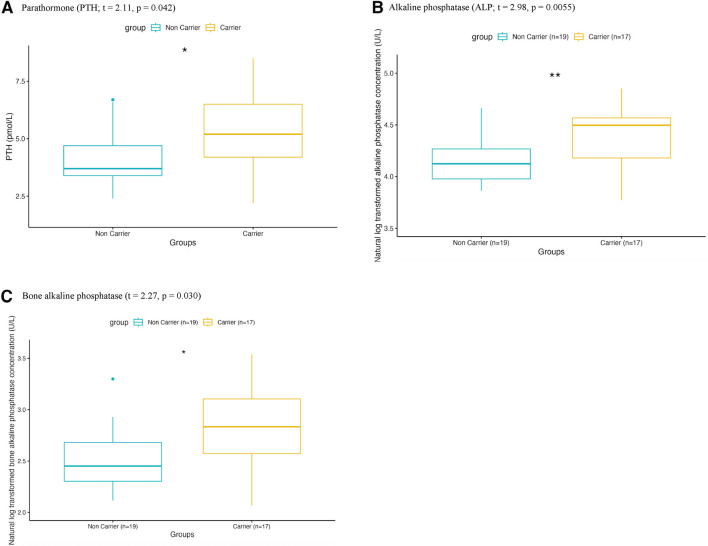
Difference in phosphocalcic profile markers concentration in serum in carriers of the c.262delG variant in the *CYP27B1* gene compared to non-carriers. Boxplots showing the results of t-test with a significant difference between the non-carrier reference group (blue; n = 19) and the carrier group (yellow; n = 17) for **(a)** parathormone **(b)** alkaline phosphatase and **(c)** bone alkaline phosphatase. Legend: *p < 0.05; **p < 0.01.

We then determined whether carrying the *CYP27B1* c.262delG variant might impact vitamin D metabolism and the phosphocalcic profile. As shown in Figure 1 and Table 2, we found significant differences between carriers and non-carriers for a) PTH (t = 2.11, p = 0.042), b) ALP (t = 2.98, p = 0.0055), and c) BAP (t = 2.27, p = 0.030), all of which had higher mean concentration in carriers as compared to non-carriers. However, after applying Benjamini–Hochberg multiple-testing adjustment, only ALP remained significant (p_adj_ = 0.044), as shown in Table 2 and Supplementary Figure S2.

**TABLE 2:** Comparison of vitamin D and phosphocalcic profile of carriers and non-carriers of the c.262delG variant in the *CYP27B1* gene and reference values.

Characteristics	Non-carriers (n = 19)		Carriers (n = 17)		Mean difference (95% CI), effect size (Hedge’s g)	Two-sided student’s t-test p-value	Adjusted p-value (Benjamini–Hochberg)	Reference values
	Mean ± standard deviation	Range	Mean ± standard deviation	Range				
Ionized calcium (mmol/L)	1.24 ± 0.04	1.15–1.31	1.25 ± 0.05	1.18–1.38	0.01 (−0.03–0.04) g = 0.07	p = 0.81	p = 0.81	1.15–1.35
Inorganic phosphorus (mmol/L)	1.21 ± 0.17	0.94–1.62	1.14 ± 0.13	0.94–1.36	0.07 (−0.17–0.04) g = −0.41	p = 0.50	p = 0.67	0.81–1.45
Parathormone (pmol/L)	4.08 ± 1.36	2.40–6.70	5.35 ± 1.66	2.20–8.50	1.25 (0.24–2.29) g = 0.82	p = 0.042*	p = 0.11	1.30–9.30
Alkaline phosphatase (U/L)	62.58 ± 16.10	33.15–106.15	86.87 ± 26.07	43.46–127.98	24.29 (9.78–38.79) g = 1.11	p = 0.0055**	p = 0.044*	42–121
Bone alkaline phosphatase (ug/L)	12.76 ± 4.55	8.20–27.10	18.10 ± 7.78	7.90–34.40	5.32 (1.06–9.58) g = 0.83	p = 0.030*	p = 0.11	Premenopausal women 4.0–14.3 μg/L Postmenopausal women 7.5–22.4 μg/L Men 7.0–20.1 μg/L
25(OH) vitamin D (nmol/L)	77.42 ± 12.62	51.50–104.50	90.43 ± 23.92	48.80–151.50	13.01 (0.25–25.76), g = 0.68	p = 0.057†	p = 0.11	75–250
Calcitriol (pmol/L)	84.68 ± 29.56	35.00–151.00	93.35 ± 21.39	55.00–126.00	8.67 (−9.00–26.33) g = 0.33	p = 0.73	p = 0.81	40–190
FGF-23 (ng/mL)	24.80 ± 29.14	0.55–75	17.81 ± 17.68	0.33–75	7.00 (−26.31–12.32) g = −0.24	p = 0.25	p = 0.40	NA

^†^p < 0.1; *p < 0.05; **p < 0.01.

To validate the robustness of these results, a sensitivity analysis was performed using ANCOVA models including (1) age, (2) sex, (3) BMI, (4) a combined model including age, sex, and BMI as covariates and (5) vitamin D consumption and sun exposure/protection data. Overall, the results remained unchanged (direction and strength (p-value) of the associations) after correction for these potential cofounders (Supplementary Table S2). A sex-stratified analysis was also conducted to account for biological and behavioral differences between females and males in vitamin D metabolism (Wierzbicka and Oczkowicz, 2022). Overall, the direction of the associations remained the same in both sexes separately, although the differences remained statistically significant only in either females or males (Supplementary Table S3).

## Discussion

With this study, we aimed to assess whether being heterozygous for a nonsense and loss of function-associated variant (c.262delG) in the *CYP27B1* impacts vitamin D metabolism and the phosphocalcic profile. We found that PTH, ALP, and BAP were significantly increased in carriers compared to non-carriers (p < 0.05). Additionally, 25(OH)D levels tended to be slightly elevated in carriers (p = 0.056). However, calcitriol levels remained unchanged (p > 0.05), as did ionized calcium, inorganic phosphorus levels and FGF-23, suggesting a compensatory mechanism contributes to maintain calcitriol homeostasis in *CYP27B1* c.262delG carriers. The observed profile mirrors that typically seen in VDDR1A, but presents in a substantially attenuated form which remains within the clinical reference values (Acar et al., 2018).

Self-reported sun exposure and protection habits data were collected for each participant to assess their impacts on vitamin D and phosphocalcic profile and on the observed associations. Other self-reported lifestyle data potentially influencing vitamin D and phosphocalcic metabolism were also collected, including vitamin D supplementation, alcohol (Ogunsakin et al., 2016; Shankar et al., 2008), tobacco and cannabis (Yang et al., 2021; de Carvalho et al., 2022), and their practice of physical activity (Goolsby and Boniquit, 2017). Overall, no difference was found for those variables between the non-carriers and carriers of the c.262delG variant in the *CYP27B1* gene (p > 0.05), and sensitivity analysis showed no significant impact of these potential covariates on the observed difference in PTH, ALP and BAP levels between groups after correction.

**FIGURE 2:**
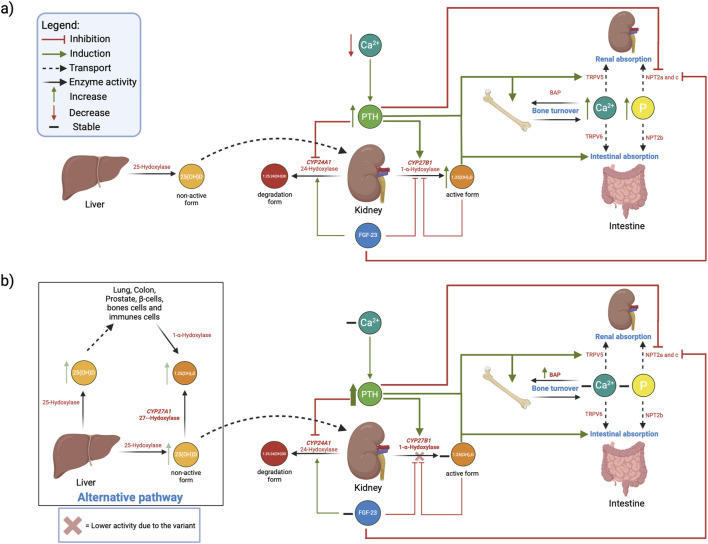
Schematic representation of the phosphocalcic metabolism and the potential impacts of carrying one allele of the c.262delG variant in the *CYP27B1* gene. Integrative figure illustrating. **(a)** The regulation of calcium, phosphorus and calcitriol by normal PTH pathway based on literature, and **(b)** Hypothetical compensatory mechanisms in people heterozygotes for *CYP27B1* c.262delG. The slight increase of PTH and 25(OH)D may enhance CYP27B1 expression and prevent decreased 1,25(OH)_2_D, and Ca^2+^ and P. Created in BioRender. Desgagné, V. (2026) https://BioRender.com/g2j9s9i. Abbreviations: 1,25(OH)_2_D, Calcitriol; 1,25-24(OH)_3_D, 1,25,24-trihydroxyvitamin D_3_; 25(OH)D, 25-hydroxyvitamin D; BAP, Bone alkaline phosphatase; Ca^2+^, Ionized calcium; *CYP27A1*, Cytochrome P450 Family 27 Subfamily A Member 1; *CYP27B1*, Cytochrome P450 Subfamily 27 Family B Member 1; FGF-23, Fibroblast growth factor 23; NPT2a/b/c, Sodium-dependent phosphate transporter type II a/b/c; P, inorganic phosphorus; PTH, Parathormone; TRPV5/6, Transient Receptor Potential Vanilloid 5/6.

Figure 2 illustrates the phosphocalcic metabolism based on literature (2a) and a hypothetical compensatory mechanism to maintain normal calcitriol, ionized calcium and inorganic phosphorus concentrations in heterozygous carriers for the *CYP27B1* c.262delG variant based on our observations (2b). Elevated PTH levels observed in individuals carrying the *CYP27B1* c.262delG variant suggest that, although calcitriol levels are normal at the time of sampling, they may not always be optimal, as increased calcitriol normally suppresses PTH synthesis through negative feedback regulation (Demay et al., 1992). This elevation in PTH, along with a slight increase in 25(OH)D substrate, may explain why calcitriol levels remain normal despite carrying a non-functional allele of the *CYP27B1* gene. As represented in Figure 2a, the PTH pathway maintains calcium and phosphorus levels by regulating bone turnover, as well as intestines absorption and renal reabsorption (Portales-Castillo and Simic, 2022; Brenda and DeLuca, 2000). Another important function of PTH reported in literature is to regulate the calcitriol levels by stimulating the 1α-hydroxylase synthesis directly via the regulation the *CYP27B1* gene’s transcription (Fraser and Kodicek, 1970; Brunette et al., 1978; Shankar et al., 2008). Hence, the slight increase of PTH and 25(OH)D we observed in c.262delG carriers may enhance *CYP27B1* expression until 1-α-hydroxylase activity is strong enough to counterbalance the synthesis of a non-functional messenger RNA and to maintain calcitriol, ionized calcium and inorganic phosphorus levels in homeostasis (Figure 2b). This hypothetical compensatory mechanism has been demonstrated previously in *Cyp27b1* knockout (KO) mice (Meyer and Pike, 2020). Other studies in humans and mice demonstrated that PTH is also implicated in the inhibition of calcitriol catabolism pathway via the post-transcriptional inhibition of *CYP24A1*, an enzyme responsible for the inactivation and degradation of calcitriol (Jeon and Shin, 2018; Jones et al., 2012). Another study in *Cyp27b1* KO mice showed that other enzymes are possibly involved in 25(OH)D catalysis and that calcitriol could be recovered from 25(OH)D by the *Cyp27a1* gene, coding for the sterol 27-hydroxylase enzyme (Nishikawa et al., 2019). Characterizing the 1-α-hydroxylase, sterol 27-hydroxylase and CYP24A enzymatic activities in *CYP27B1* c.262delG carriers and how they cope with loss of activity is needed for better understanding those compensatory mechanisms.

Interestingly, the increased levels of bone turnover (Vimalraj, 2020) ALP and BAP markers we found also suggest active bone resorption and formation in individuals heterozygous for the *CYP27B1* c.262delG variant. Indeed, elevated PTH stimulates bone resorption, mostly, and formation to maintain phosphocalcic homeostasis (Liu et al., 2024; Zhao et al., 1999), which was observed in our results with unchanged ionized calcium and inorganic phosphorus levels. However and since increased bone remodeling has been shown to affect bone health (Desoutter et al., 2012), further investigation is needed to better understand the impact of elevated PTH in heterozygous for a *CYP27B1* variant. This is however reassuring at this point that carriers and non-carriers have similar bone mass. Although individuals heterozygous for autosomal recessive conditions are generally asymptomatic (Yıldırım et al., 2022), studies suggest a possible spectrum of milder subclinical phenotypes in heterozygous carriers of certain diseases (Kalyta et al., 2023). Depending on the extent of these impacts, individuals heterozygotes for the c.262delG variant in the *CYP27B1* gene may be at higher risk of osteoporosis and other diseases on the long term because of their slightly affected vitamin D metabolism (Tissandié et al., 2006). Further research is needed to address this hypothesis.

### Strengths and limitations

One of the strengths of the study is that the participants were recruited among a genetically well characterized population known for its founder effect (Bchetnia et al., 2021). This founder effect is associated with a high carrier rate of VDDR1A (1/27), allowing for the investigation of a large (in the context of rare disease) number of carriers and in addition of the same pathogenic variant. The recruitment period was also short (about 6 weeks), and all participants were recruited during the same season (July and August 2023), limiting the impact of sun exposure on vitamin D and phosphocalcic profile. Another strength of our study is the comprehensive biochemical characterization in carriers and non-carriers, including the bone turnover markers BAP and FGF-23, allowing the robust comparison of key markers of vitamin D and phosphocalcic metabolism. Our study also has limitations. Although large for a study on a rare genetic disorder, the cohort size remains relatively small (n = 36), which results in a lack of statistical power to detect small effect sizes for some of the tested variables. The small cohort also included family members, research staff and their relatives, which may have introduced a selection bias and limited the generalizability of the results. Also, this study is cross-sectional and participants relatively young, which does not allow for assessment of long-term impacts of these alterations on bone health or risk to develop vitamin-D-related conditions. Another limitation is that we only measured bone mass and not density, which would have allowed us to assess the risk of osteoporosis. Finally, our study design did not allow us to measure 1α-hydroxylase, sterol 27-hydroxylase and CYP24A enzymatic activities, which could have improved our understanding, mechanistically, of the impact of carrying the *CYP27B1* c.262delG variant.

## Conclusion

We characterized the impact of being heterozygous for the *CYP27B1* c.262delG variant on vitamin D metabolism and phosphocalcic homeostasis. Levels of PTH, ALP and BAP were higher in carriers compared to non-carriers but remained within clinical reference values, while calcitriol, ionized calcium, and inorganic phosphorus remained similar between the two groups. Although the increase in 25(OH)D did not reach statistical significance, it tended toward higher levels in carriers. These findings, observed in asymptomatic individuals, suggest that the phosphocalcic profile is slightly affected in carriers of the c.262delG variant, although it remains within the clinical reference values. Further studies are needed to investigate the impact of slightly elevated PTH and active bone remodelling on bone health, as impaired phosphocalcic profile and vitamin D metabolism has been shown to be implicated in bone diseases such as osteoporosis and other metabolic conditions.

## Data Availability

The original contributions presented in the study are included in the article/Supplementary Material, further inquiries can be directed to the corresponding author.

## Abbreviations

25(OH)D, 25-hydroxyvitamin D; ALP, Alkaline phosphatase; ANCOVA: Analysis of covariance; BAP, Bone alkaline phosphatase; BMI: Body mass index; Calcitriol, 1,25-dihydroxyvitamin D; CHUM, *Centre hospitalier de l’Université de Montréal*; CIUSSS-SLSJ, *Centre Intégré Universitaire de Santé et de Services Sociaux du Saguenay–Lac-Saint-Jean*; *CYP27B1*, Cytochrome P450 Subfamily 27 Family B Member 1; FGF-23, Fibroblast growth factor 23; HDL-C, High-density lipoprotein cholesterol; ICF, Information and consent form; KO, Knockout; LDL-C, Low-density lipoprotein cholesterol; PTH, Parathormone; SD, Standard deviation; SLSJ, Saguenay–Lac-Saint-Jean; TG, Triglyceride; Total-C, Total cholesterol; VDDR1A, Vitamin D-dependent rickets type 1A.
