# Supplementary figures and images for: Heterozygous CYP27B1 c.262delG pathogenic variant and its impact on vitamin D metabolites and phosphocalcic profile in humans
Source: Front Physiol. 2026 Jan 7;16:1716877. doi: 10.3389/fphys.2025.1716877 (PMC12820427; doi:10.3389/fphys.2025.1716877)

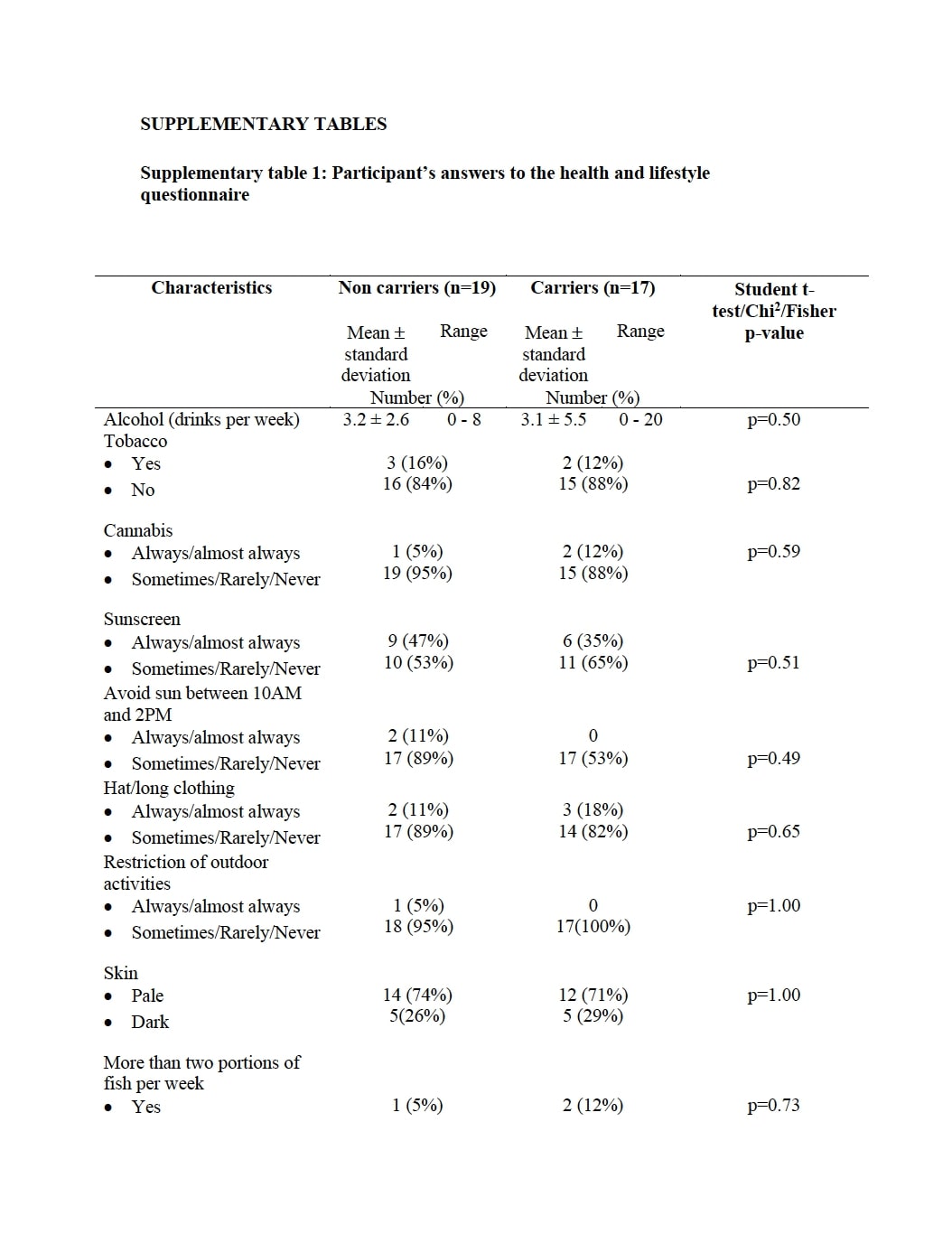


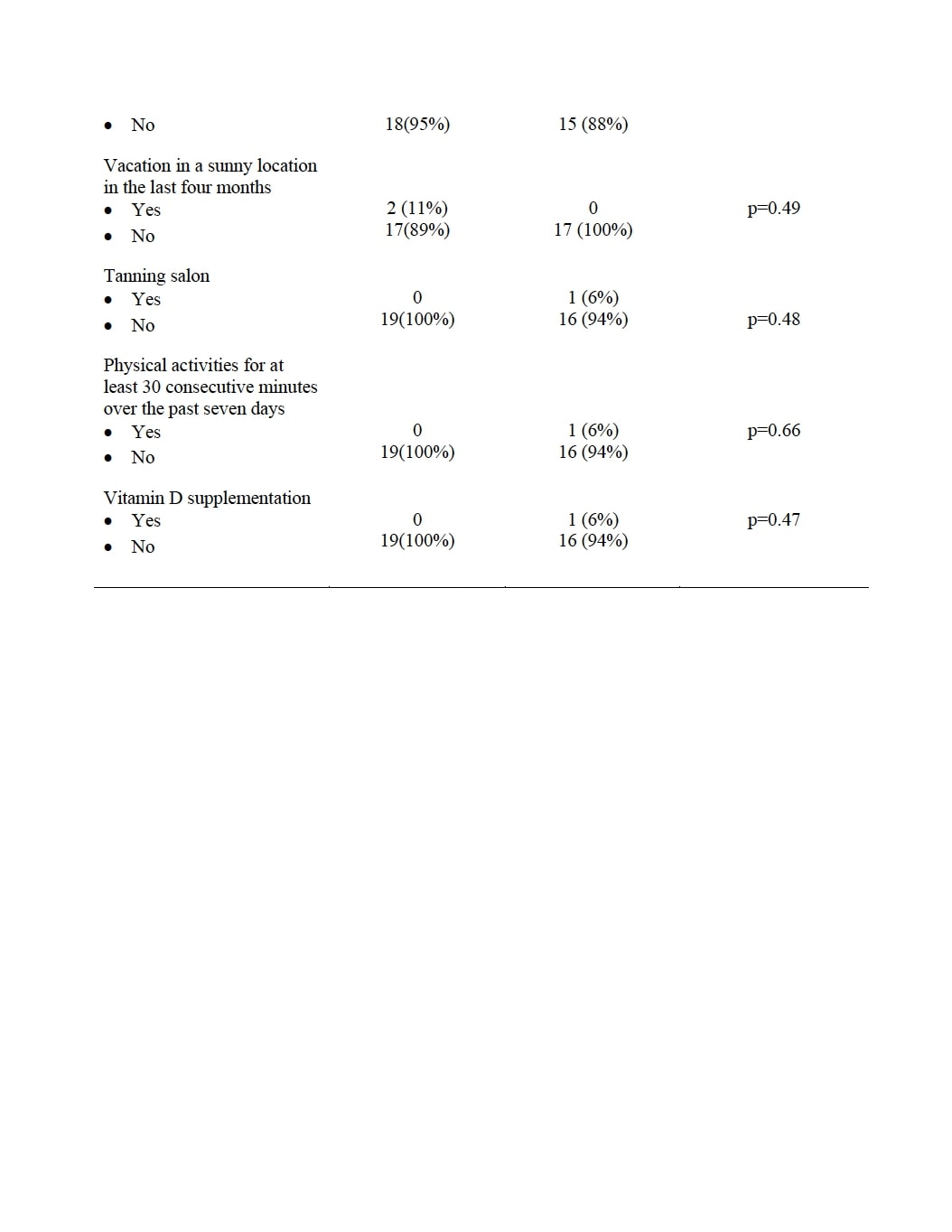


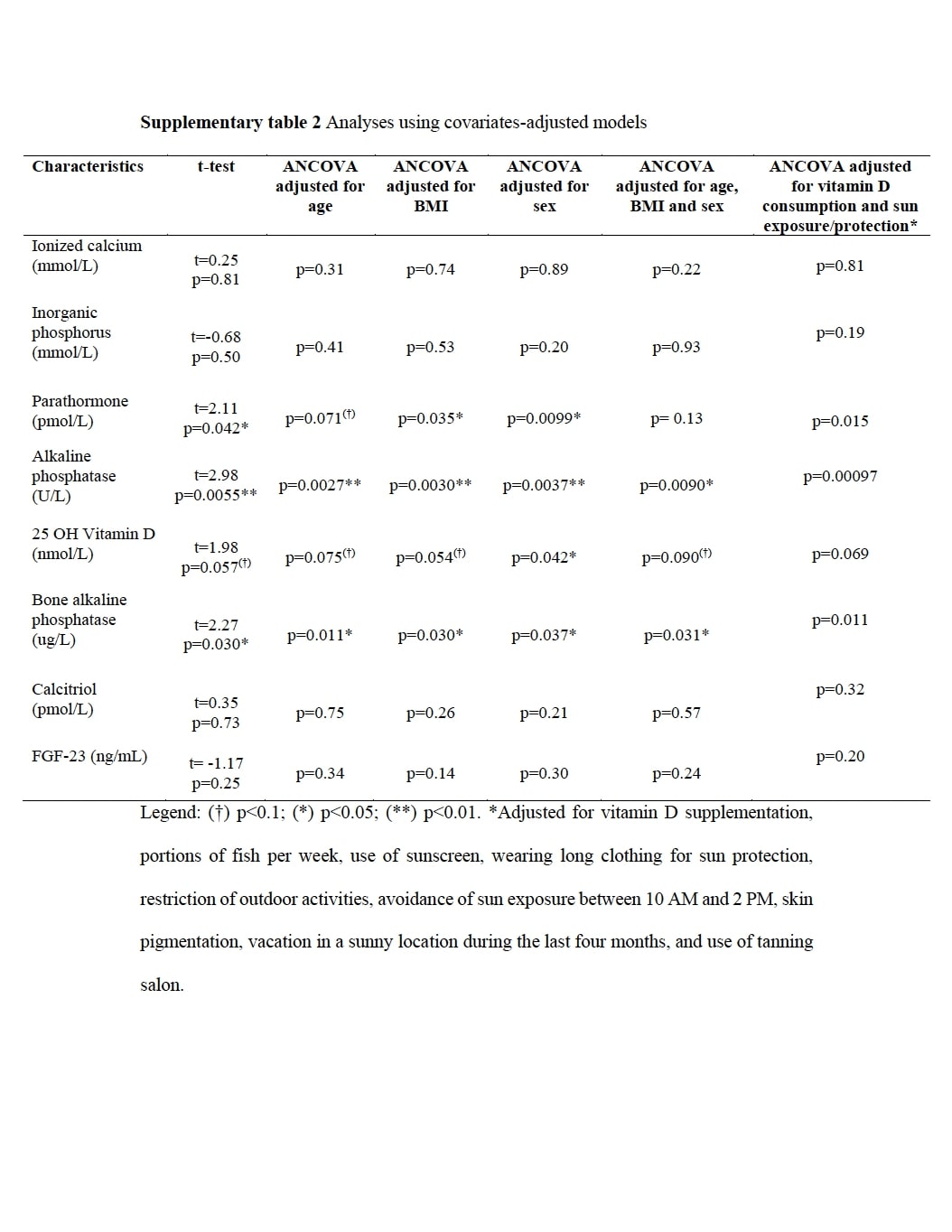


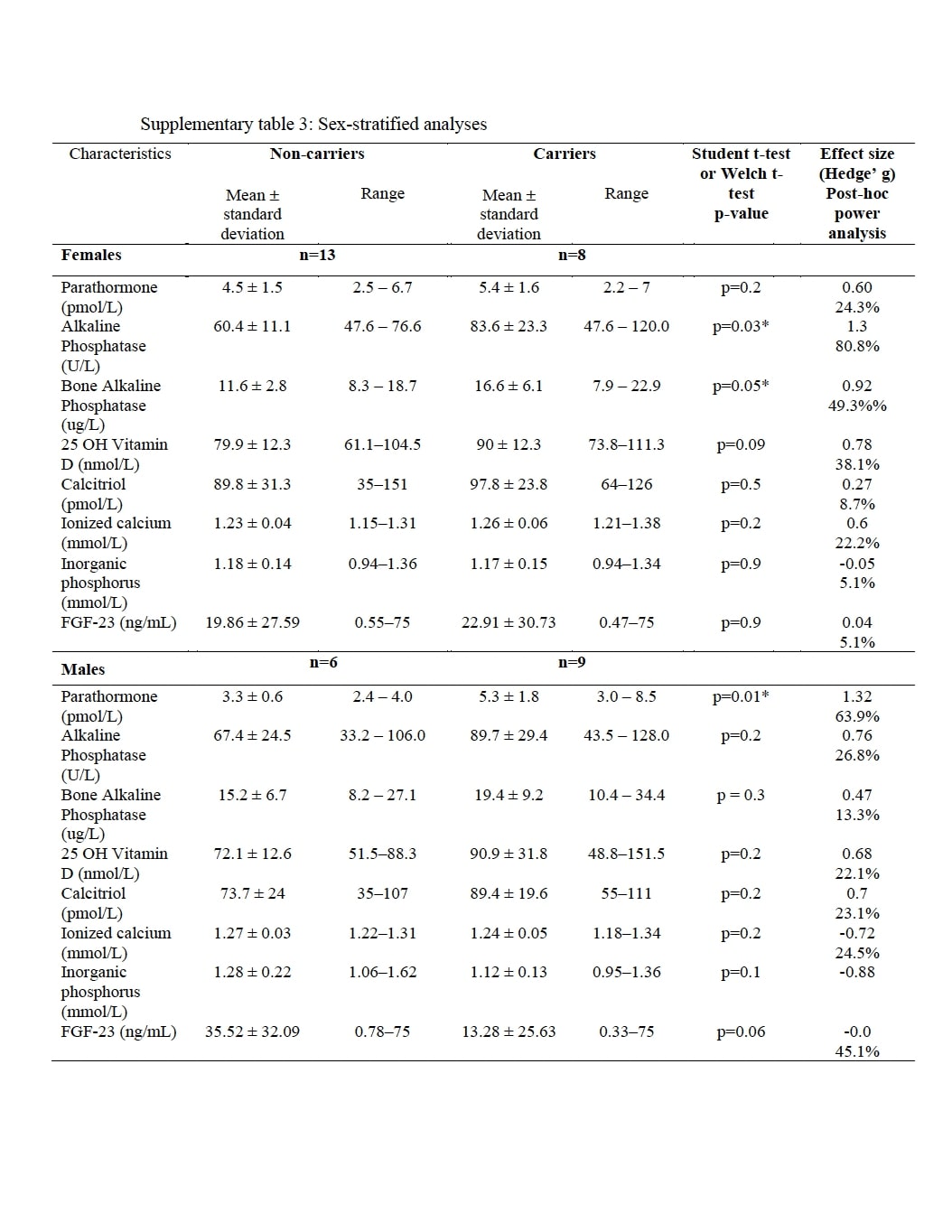


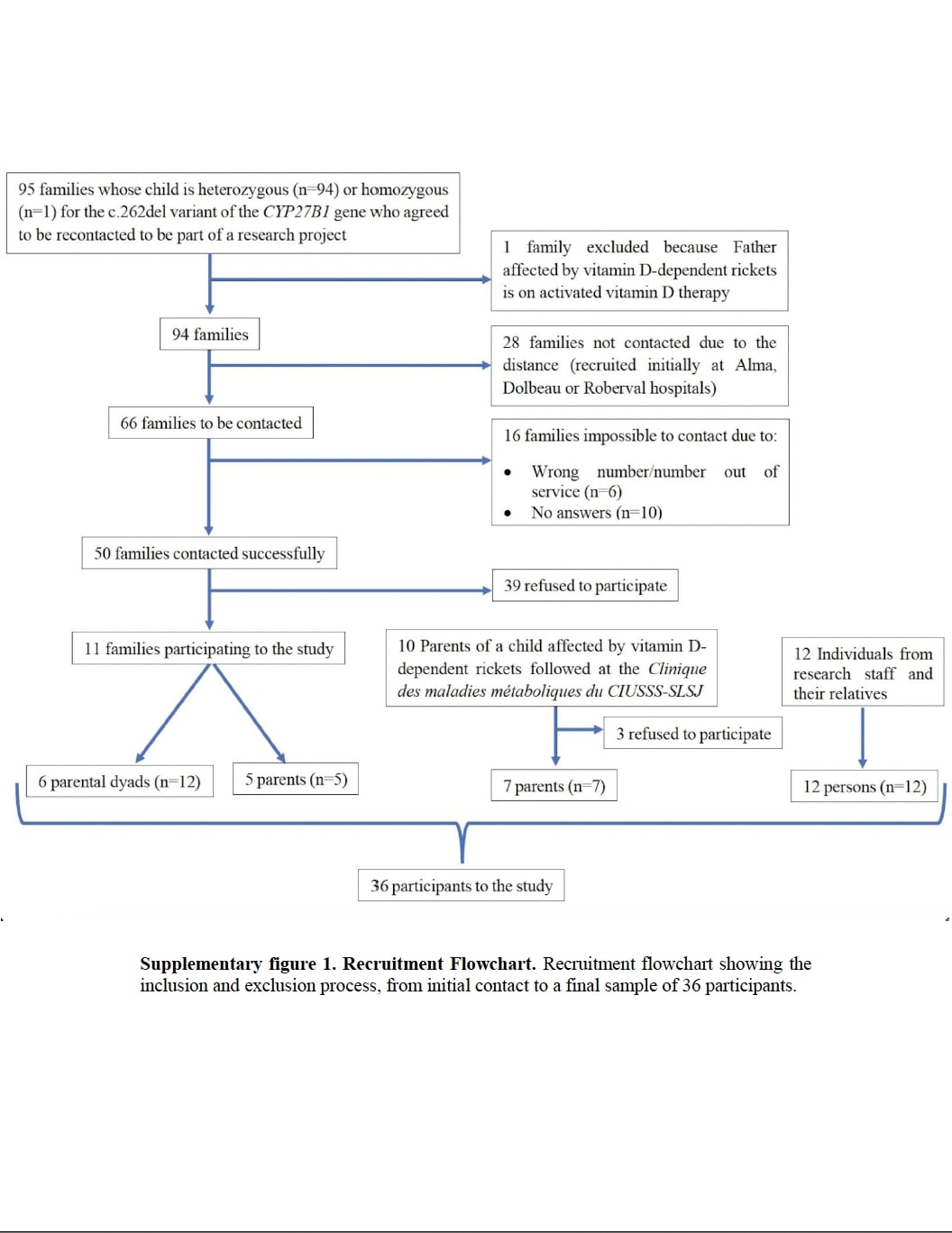


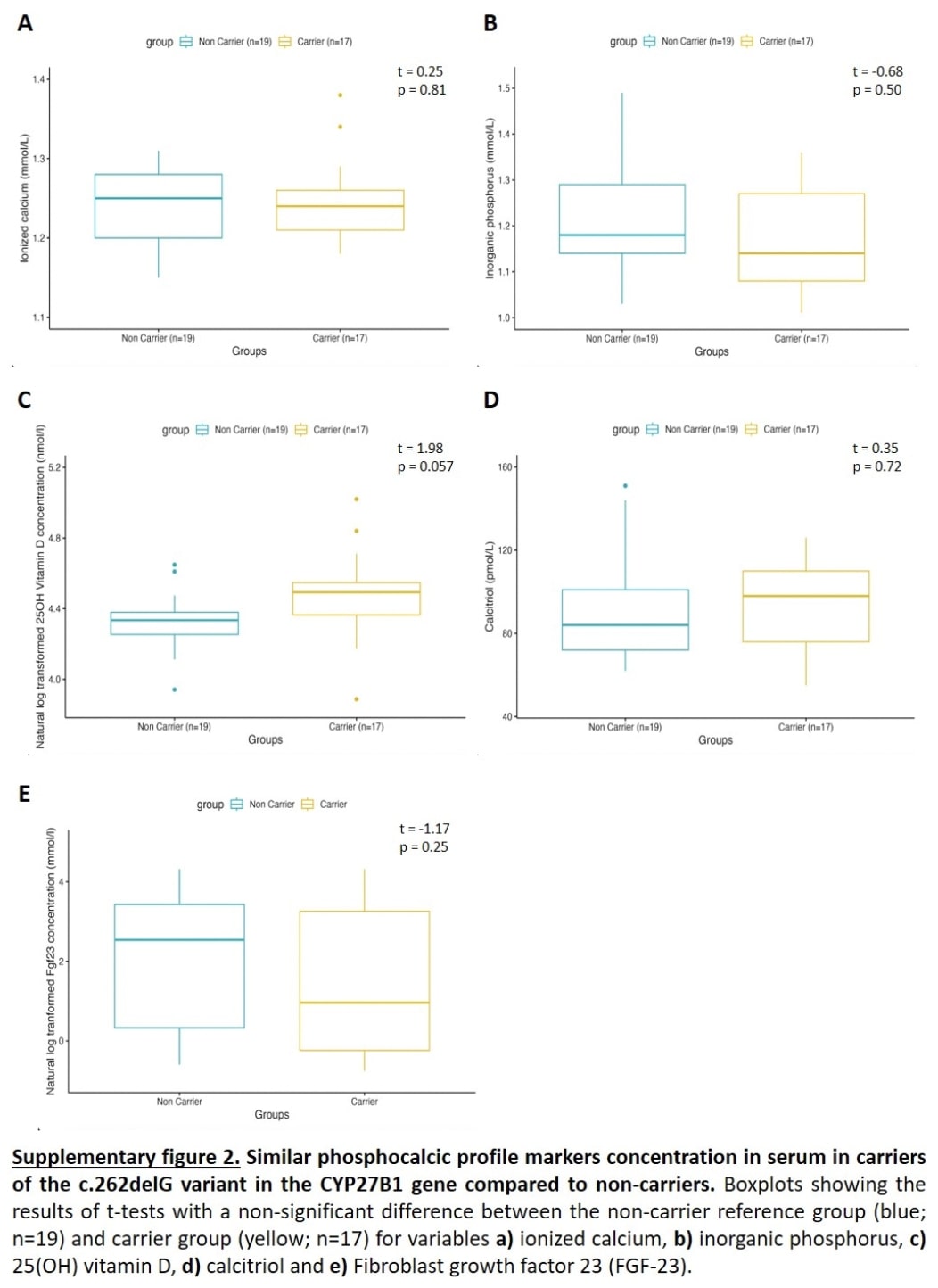

Supplement: Supplementary file 1 [file Supplementaryfile1.docx]
